# Morphometric Inverse Divergence Networks Combined with HYDRA Identify Parkinson’s Disease Subtypes with Distinct Transcriptomic and Serum Biomarker Profiles

**DOI:** 10.3390/ijms27156769

**Published:** 2026-07-28

**Authors:** Maria Giovanna Bianco, Camilla Calomino, Maria Celeste Bonacci, Maria Eugenia Caligiuri, Costanza Maria Cristiani, Luana Scaramuzzino, Jolanda Buonocore, Pier Paolo Arcuri, Aldo Quattrone, Andrea Quattrone

**Affiliations:** 1 Neuroscience Research Center, University “Magna Graecia”, 88100 Catanzaro, Italy; camilla.calomino@unicz.it (C.C.); mariaceleste.bonacci@unicz.it (M.C.B.); me.caligiuri@unicz.it (M.E.C.); costanza.cristiani@unicz.it (C.M.C.); scaramuzzino.luana@unicz.it (L.S.); jolanda.buonocore@unicz.it (J.B.); pierpaoloarcuri@unicz.it (P.P.A.); quattrone@unicz.it (A.Q.); an.quattrone@unicz.it (A.Q.); 2 Department of Medical and Surgical Sciences, Magna Graecia University, 88100 Catanzaro, Italy; 3 Institute of Neurology, Department of Medical and Surgical Sciences, Magna Graecia University, 88100 Catanzaro, Italy

**Keywords:** Parkinson’s disease, morphometric inverse divergence (MIND), cortical networks, disease subtypes, imaging transcriptomics, neurofilament light chain (NfL), glial fibrillary acidic protein (GFAP)

## Abstract

Parkinson’s disease (PD) is the second most common age-related neurodegenerative disorder, yet it remains unclear whether cortical architecture can reveal biologically distinct subtypes with distinct molecular and serum biomarker signatures. Two hundred PD patients and 121 healthy controls underwent structural MRI. Subject-specific cortical similarity networks were constructed using Morphometric INverse Divergence (MIND), and subtypes were identified with HYDRA. Spatial patterns were linked to regional gene expression from the Allen Human Brain Atlas through partial least squares regression, followed by functional and cell-type enrichment analyses. Serum neurofilament light chain (NfL) and glial fibrillary acidic protein (GFAP) were quantified using single-molecule array assays. No significant MIND differences emerged when PD patients were analysed as a single group. HYDRA identified two subtypes (ARI = 0.85) with divergent cortical organization that only partially overlapped with conventional motor phenotypes. Cluster 1 exhibited temporo-parietal MIND increases associated with synaptic and oligodendroglial signatures, without serum biomarker associations. Cluster 2 showed widespread fronto-cingulate MIND reductions enriched for mitochondrial, lysosomal, and proteostatic pathways, including the KEGG Parkinson’s disease pathway, and these reductions correlated with higher serum NfL and GFAP. These findings reveal two biologically distinct PD subtypes with divergent molecular architecture and systemic neurodegeneration beyond conventional motor phenotyping.

## 1. Introduction

Parkinson’s disease (PD) is the second most common age-related neurodegenerative disorder, and its prevalence is increasing markedly with population ageing. Although the diagnosis is based on a recognizable motor syndrome, patients differ widely in clinical presentation, disease progression, and the severity of non-motor and cognitive manifestations [1]. Patients are conventionally classified into motor phenotypes, most commonly the tremor-dominant (TD) and postural instability/gait difficulty (PIGD) subtypes [2]. These categories, however, overlap substantially, evolve over the disease course, and have limited prognostic value [3,4], indicating that such clinical classifications capture only part of the underlying disease biology.

Neuropathologically, PD is characterized by the progressive spread of Lewy body pathology that ultimately reaches widespread cortical areas, and the degree of neocortical involvement parallels disease severity and the emergence of cognitive decline [5]. In line with these pathological findings, structural MRI studies have reported cortical thinning and gray matter loss involving temporal, frontal, parietal, and occipital regions [6,7,8]. However, most studies have examined cortical regions in isolation, one morphometric feature at a time, an approach that does not capture the distributed and coordinated nature of cortical degeneration in PD [9].

To address this, the Morphometric INverse Divergence (MIND) framework quantifies structural similarity between cortical regions by integrating multiple vertex-wise morphometric features, generating individualized maps of cortical organization [9]. Because morphometrically similar regions tend to share developmental origin, gene expression profiles, and axonal connectivity, MIND provides a biologically informed measure of cortical architecture and has recently been applied across several neurological and psychiatric disorders [10,11,12,13]. Building on this multivariate representation, Heterogeneity Through Discriminative Analysis (HYDRA) provides a semi-supervised framework for identifying patient subgroups by modeling multivariate deviations from healthy controls [14,15,16]. When combined with MIND-derived measures, this approach enables the investigation of disease heterogeneity beyond conventional clinical classifications.

The spatial distribution of neurodegeneration in PD is non-uniform, with cortical atrophy preferentially affecting regions enriched in synaptic-related genes [17]. Integrative imaging–transcriptomic studies have linked large-scale neuroanatomical alterations to region-specific gene expression signatures implicated in PD pathogenesis [18,19]. In this context, mapping subtype-specific patterns of cortical degeneration onto regional transcriptomic data from the Allen Human Brain Atlas (AHBA) offers an opportunity to investigate whether subtype-specific cortical alterations are spatially constrained by regional gene expression gradients, and to link macroscale network organization to the molecular pathways implicated in regional vulnerability in PD. Whether these subtype-specific network and transcriptomic signatures are accompanied by measurable differences in systemic markers of neurodegeneration remains an open question. Neurofilament light chain (NfL) and glial fibrillary acidic protein (GFAP) have emerged as promising serum markers of axonal injury and astrocytic activation, respectively, and have been associated with disease severity and progression in PD [20,21,22]. Integrating these biomarkers with imaging and transcriptomic measures may help determine whether biologically distinct subtypes also differ in the degree of systemic neurodegeneration. In this study, we adopt a multiscale, data-driven framework to investigate PD heterogeneity. Previous imaging–transcriptomic studies in PD have largely treated the disorder as a single group and therefore could not address whether distinct biological subtypes contribute to its clinical variability [18,19,23].

Here, we combined individual-level MIND networks, HYDRA-based subtyping, regional transcriptomic mapping, and serum biomarkers within a single cohort to test whether distinct patterns of cortical organization define biologically relevant PD subtypes differing in molecular architecture and systemic neurodegeneration. The analytical framework followed a sequential logic. Subject-specific MIND networks were first constructed from structural MRI, and regional MIND strength profiles were then entered into HYDRA to identify data-driven PD subtypes relative to HC. Subtype-specific case–control MIND t-maps were subsequently used as imaging phenotypes in PLS regression with regional AHBA gene expression, followed by functional and cell-type enrichment analyses. In parallel, serum NfL and GFAP were examined as downstream correlates of regional MIND strength within each subtype. Thus, MIND provided the imaging features, HYDRA performed subtype identification, and transcriptomic and serum biomarker analyses provided complementary biological characterization without contributing to subtype assignment.

## 2. Results

### 2.1. Demographic and Clinical Data

A total of 200 patients with Parkinson’s disease and 121 healthy controls were included in the study. No significant differences were observed between PD patients and HC in terms of age (*p* = 0.136) or sex distribution (*p* = 0.095) (Table 1).

Within the PD cohort, HYDRA identified two clusters (Cluster 1: n = 96; Cluster 2: n = 104; ARI = 0.85). Significant differences in demographic characteristics were observed across groups (Table 2). Sex distribution differed significantly among HC and PD clusters (χ^2^, *p* = 0.0003), with Cluster 1 showing a higher proportion of males compared to Cluster 2 and HC. Age also differed across groups (*p* = 0.0046), with Cluster 2 being significantly older than HC, while Cluster 1 showed lower age compared to Cluster 2.

Regarding clinical features, tremor scores were significantly higher in Cluster 1 compared to Cluster 2 (*p* = 0.026), whereas no differences were observed for PIGD scores (*p* = 0.256). No significant differences between clusters were found for MDS-UPDRS motor scores (*p* = 0.892), MDS-UPDRS total scores (*p* = 0.629), or Hoehn and Yahr stage (*p* = 0.130). Disease onset was significantly earlier in Cluster 1 compared to Cluster 2 (*p* = 0.030), while disease duration (*p* = 0.306) and levodopa equivalent daily dose (LEDD) (*p* = 0.461) did not differ between clusters. No significant differences were observed in serum biomarker levels (Aβ42/Aβ40 ratio, p-tau217, GFAP, NfL) across HC and PD clusters (all *p* > 0.05), although a trend toward higher NfL levels was observed in Cluster 2 (*p* = 0.081).

### 2.2. MIND Strength Differences Across PD Clusters

Prior to subtype stratification, case–control comparisons between the entire PD cohort and healthy controls did not reveal any significant regional differences in MIND strength after permutation-based FWER correction (*p* < 0.05). Following the identification of PD subtypes using the HYDRA framework, distinct and opposing patterns of structural similarity emerged between clusters.

**Figure 1:**
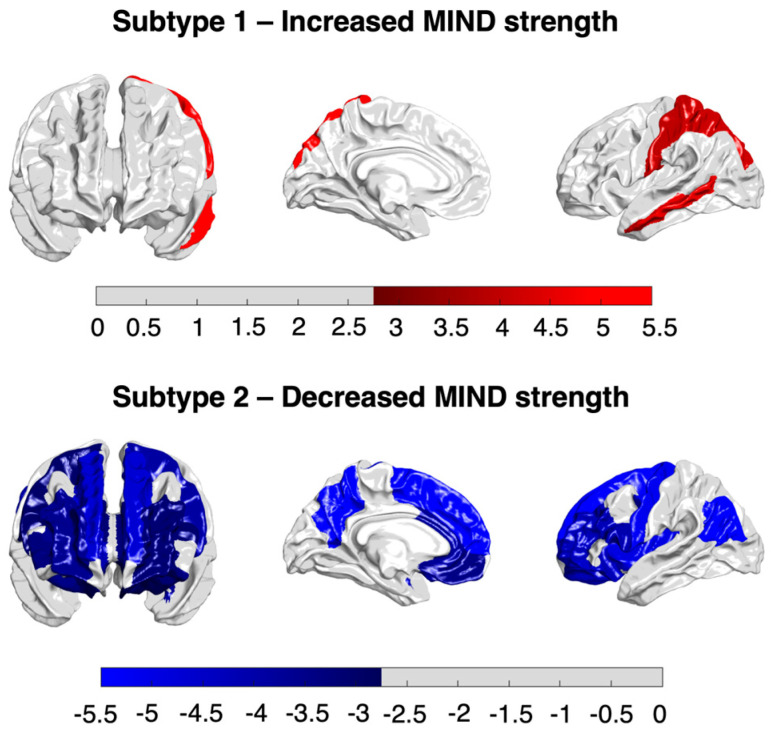
Case–control differences in MIND network strength across PD subtypes. Cortical surface maps display regions with statistically significant differences in morphometric similarity (MIND) strength between each PD subtype and healthy controls (FWER-corrected, *p* < 0.05). Three views are shown for each subtype: dorsal, medial left hemisphere (LH), and lateral LH. Color intensity reflects the magnitude of the t-statistic. (Top) PD Subtype 1 exhibited focal increases in MIND strength (red scale; 6 regions) involving bilateral temporal cortex (lh/rh middle temporal, rh superior temporal), left superior parietal and postcentral cortex, and right pars orbitalis (t-range: 3.55–4.56). (Bottom) PD Subtype 2 showed widespread decreases in MIND strength (blue scale; 32 regions) encompassing bilateral frontoparietal regions (superior frontal, inferior parietal, supramarginal, precentral, rostral middle frontal, caudal middle frontal, pars opercularis, pars triangularis), anterior and posterior cingulate cortex, orbitofrontal cortex (lateral, medial, pars orbitalis), precuneus, insula, and lateral occipital cortex (t-range: −3.38 to −7.37).

Group-averaged MIND strength across cortical regions is illustrated in Figure 1. At the whole-brain level, Cluster 1 exhibited localized increases in MIND strength relative to HC, whereas Cluster 2 showed a widespread reduction in MIND strength (Figure 1, Supplementary Table S1).

In detail, Cluster 1 demonstrated significantly higher MIND strength compared to HC in a limited set of regions, primarily involving bilateral temporal and parietal cortices, including the middle temporal, superior parietal, and postcentral regions, as well as the right superior temporal and pars orbitalis cortices.

In contrast, Cluster 2 exhibited extensive reductions in MIND strength relative to HC, affecting a broad set of cortical regions. These alterations prominently involved bilateral frontal cortices, including the superior frontal, rostral and caudal middle frontal, and orbitofrontal regions, as well as cingulate areas (rostral, caudal, and posterior divisions). Additional decreases were observed in parietal regions (inferior parietal, supramarginal, and precuneus), as well as in the insula, precentral cortex, and lateral occipital areas. To further characterize the spatial organization of these alterations, regional values were summarized across canonical functional networks (Yeo 7 networks) and cytoarchitectonic classes (von Economo atlas).

Across Yeo functional networks, Cluster 1 showed a consistent pattern of increased MIND strength relative to healthy controls, particularly within the somatomotor, dorsal attention, and visual networks. In contrast, Cluster 2 exhibited reduced MIND strength across multiple networks, with the most pronounced decreases observed in the default mode and frontoparietal networks. Limbic and salience networks showed intermediate patterns, with partial overlap between the two subtypes (permutation FDR-corrected *p* < 0.05; Supplementary Figure S1, superior panel).

A similar divergence was observed across von Economo cytoarchitectonic classes. Cluster 1 demonstrated higher MIND strength in associative cortical classes, including granular and association cortices, whereas Cluster 2 showed widespread reductions across these same classes, particularly within agranular and frontal regions. Primary sensory areas exhibited relatively smaller differences between clusters (permutation FDR-corrected *p* < 0.05; Supplementary Figure S1, inferior panel).

### 2.3. Transcriptomic Correlates of MIND Alterations in PD Subtypes

**Figure 2:**
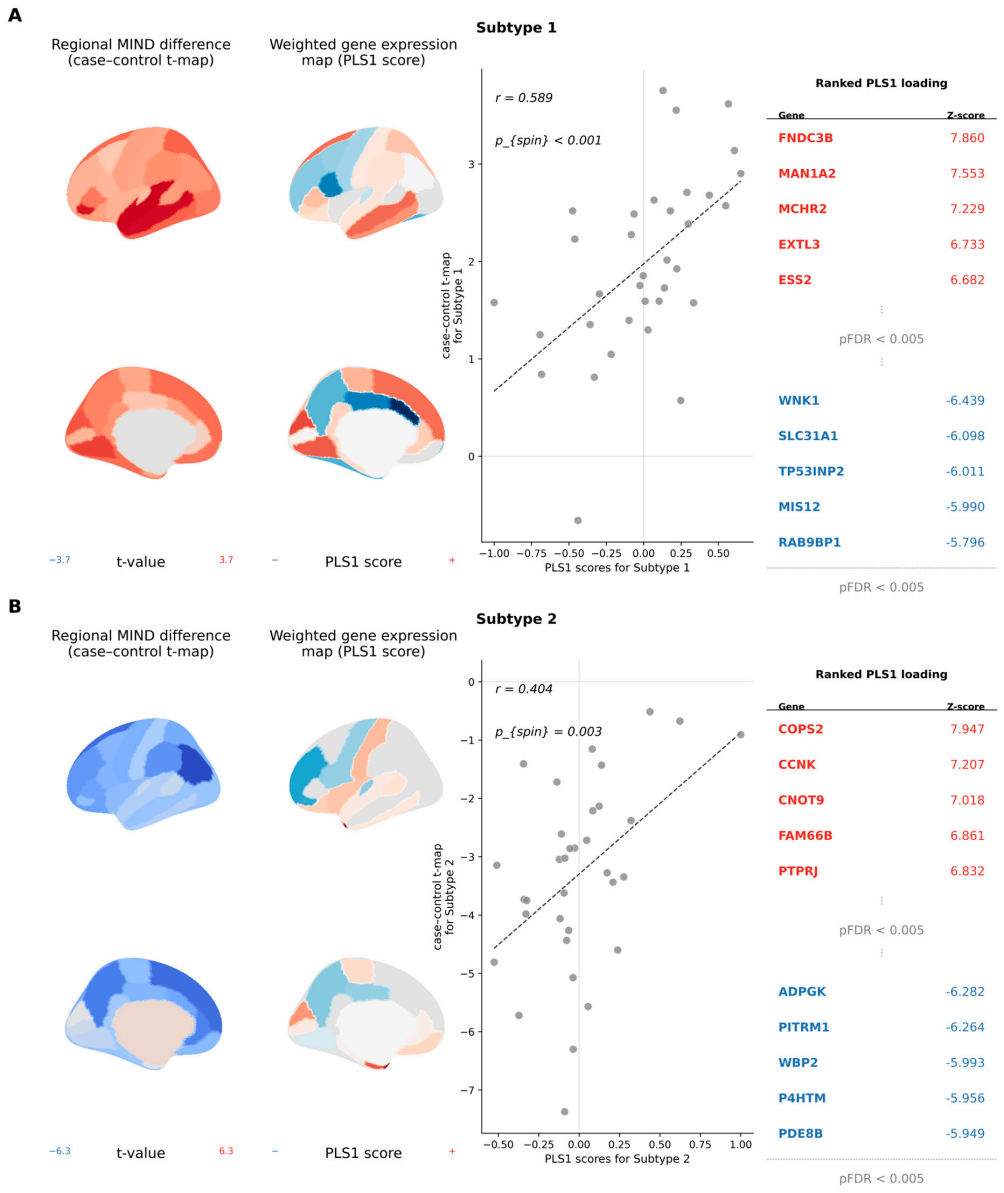
**Transcriptomic correlates of MIND alterations in PD subtypes.** (**A**) Subtype 1 (Cluster 1 vs. HC). Left: cortical surface map of case–control t-values reflecting regional MIND strength differences (Cluster 1 > HC, warm colors). Center-left: weighted gene expression map derived from partial least squares (PLS1) regression, showing the spatial distribution of PLS1 scores across 68 cortical regions. Center-right: scatter plot showing the spatial correlation between empirical MIND t-values and PLS1-predicted scores (r = 0.589, p-spin < 0.001), with each dot representing one cortical region. Right: ranked PLS1 gene loadings (Z-scores from bootstrap resampling, 5000 iterations), with top positively weighted genes shown in red and top negatively weighted genes in blue (threshold: |Z| > 3.0, FDR < 0.005). (**B**) Subtype 2 (Cluster 2 vs. HC). Same layout as Panel A. Cortical surface map reflects MIND reductions in Cluster 2 (cool colors). PLS1 scores align with the spatial pattern of MIND reductions (r = 0.404, p-spin = 0.003). Top positively and negatively weighted genes are shown with corresponding Z-scores. PLS, partial least squares; HC, healthy controls; FDR, false discovery rate.

To identify molecular signatures associated with subtype-specific MIND alterations, partial least squares (PLS) regression was applied using regional case–control t-statistic maps as imaging phenotypes (Figure 2).

The first latent component (PLS1) was statistically significant in both subtype comparisons (spin test, 10,000 rotations, *p* < 0.05), capturing the maximal covariance between cortical gene expression and MIND alterations. PLS1 explained 28.9% of the variance in Cluster 1 and 20.3% in Cluster 2.

The spatial distribution of PLS1 scores showed significant correspondence with empirical MIND t-maps in both subtypes (Cluster 1: Spearman’s r = 0.589, p-spin < 0.001; Cluster 2: r = 0.404, p-spin = 0.003; Figure 2), indicating a robust alignment between transcriptomic patterns and structural network alterations.

Following bootstrap resampling (5000 iterations), gene weights were normalized to Z-scores and thresholded at |Z| > 3. This yielded 675 positively weighted and 410 negatively weighted genes in Cluster 1, and 764 positively weighted and 395 negatively weighted genes in Cluster 2 (Figure 2). PLS1-positive (PLS1+) genes represent transcripts positively associated with the spatial pattern of MIND alterations, whereas PLS1-negative (PLS1−) genes show an inverse association. The two subtypes were characterized by distinct PLS1 cortical gradients, oriented along a posterior-to-anterior axis in Cluster 1 and a limbic-to-prefrontal axis in Cluster 2.

To assess disease relevance, genes with robust PLS1 weights (|Z| > 3) were intersected with the Allen Human Brain Atlas “Parkinson disease” gene classification. For each overlapping gene, Spearman correlation was computed between its regional expression profile and the cluster-specific case–control MIND t-map, followed by FDR correction (q < 0.05). This fully data-driven procedure, without a priori gene selection, retained five genes in Cluster 1 (VPS26A, PSMA4, GBA2, CYCS, and DNAJC13) and ten in Cluster 2 (Figure 2; Supplementary Table S2). Notably, DNAJC13 (PLS1+) and GBA2 (PLS1−) were consistently identified across both clusters, suggesting partially shared molecular substrates underlying distinct patterns of network reorganization.

### 2.4. Functional Enrichment Analysis

**Figure 3:**
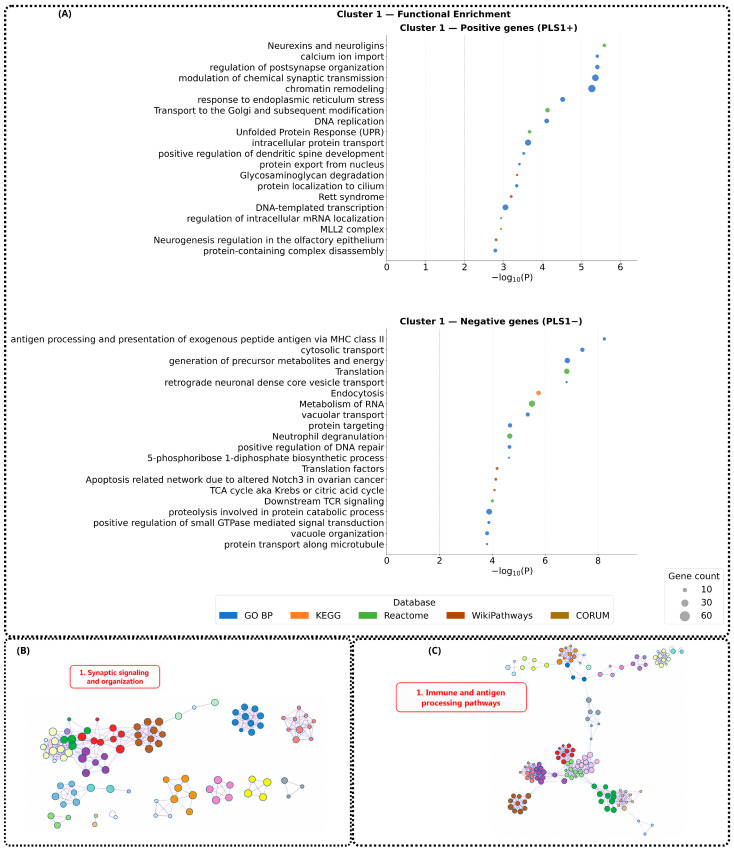
Functional enrichment network analysis of PLS1-significant gene sets across PD subtype 1. (**A**) Bubble plots showing the top enriched biological terms for Cluster 1 positive genes (PLS1+; left panel, n = 675) and Cluster 1 negative genes (PLS1−; right panel, n = 410), identified through Metascape multi-ontology enrichment analysis. The x-axis represents −log_10_(*p*-value); bubble size reflects the number of input genes annotated to each term; bubble color indicates the source database (GO Biological Process, KEGG, Reactome, WikiPathways, or CORUM). Only terms meeting a Benjamini–Hochberg FDR-corrected threshold of p-FDR < 0.05 are shown. (**B**) Network plot for Cluster 1 positive genes (PLS1+). Each node represents an enriched biological term; edges connect terms sharing pathway membership above a kappa similarity threshold of 0.3. Node color denotes cluster identity; node size reflects gene count per term. The dominant cluster reflects convergent enrichment of synaptic signaling and organization pathways, including neurexin–neuroligin interactions, regulation of postsynapse organization, modulation of chemical synaptic transmission, and chromatin remodeling. (**C**) Network plot for Cluster 1 negative genes (PLS1−). The dominant cluster reflects enrichment of immune and antigen processing pathways, including antigen processing and presentation via MHC class II, cytosolic transport, generation of precursor metabolites and energy, translational regulation, and the TCA cycle. More details are reported in Supplementary Figure S4.

To characterize the biological processes associated with subtype-specific transcriptomic signatures, functional enrichment analysis was performed on PLS1-positive and PLS1-negative gene sets using Metascape. In Cluster 1, PLS1-positive genes were significantly enriched in synaptic and plasticity-related processes, including neurexin–neuroligin signaling, postsynaptic organization, and modulation of chemical synaptic transmission (Figure 3). In contrast, PLS1-negative genes were enriched in metabolic and cellular maintenance processes, including energy production, translation, and RNA metabolism (Figure 3). These results indicate that regions exhibiting increased MIND strength in Cluster 1 are associated with transcriptional programs related to synaptic organization and plasticity, whereas regions with relatively lower similarity show enrichment in metabolic and immune-related processes.

**Figure 4:**
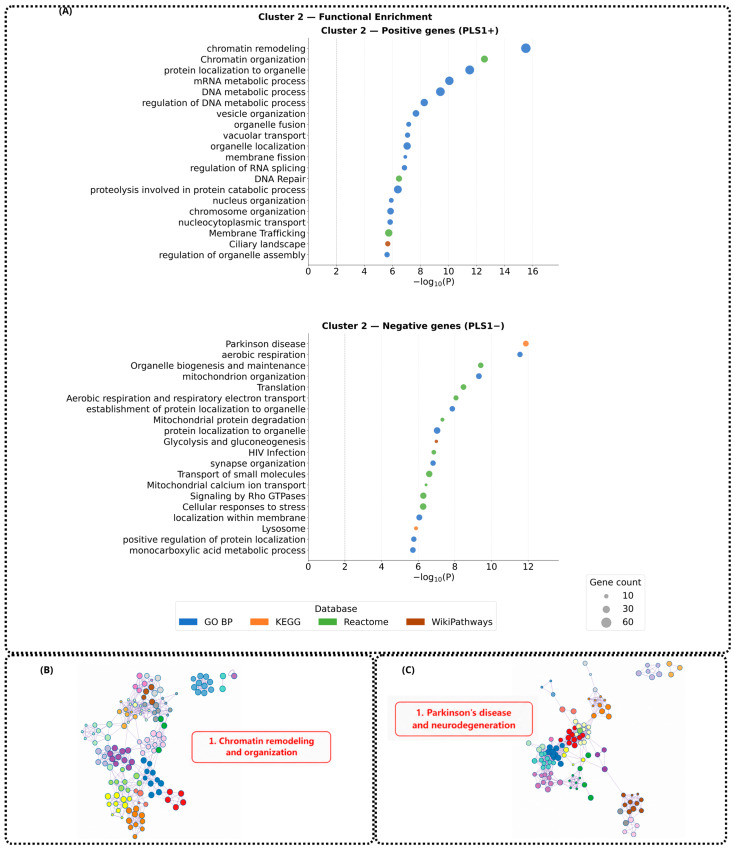
Functional enrichment network analysis of PLS1-significant gene sets across PD subtype 2. (**A**) Bubble plots showing the top enriched biological terms for Cluster 2 positive genes (PLS1+; left panel, n = 764) and Cluster 2 negative genes (PLS1−; right panel, n = 395), identified through Metascape multi-ontology enrichment analysis. The x-axis represents −log_10_(*p*-value); bubble size reflects the number of input genes annotated to each term; bubble color indicates the source database (GO Biological Process, KEGG, Reactome, WikiPathways, or CORUM). Only terms meeting a Benjamini–Hochberg FDR-corrected threshold of p-FDR < 0.05 are shown. (**B**) Network plot for Cluster 2 positive genes (PLS1+). Each node represents an enriched biological term; edges connect terms sharing pathway membership above a kappa similarity threshold of 0.3. Node color denotes cluster identity; node size reflects gene count per term. The dominant cluster reflects convergent enrichment of chromatin remodeling and organization pathways, alongside DNA and mRNA metabolic processes, vesicle organization, organelle fusion, and nucleocytoplasmic transport. (**C**) Network plot for Cluster 2 negative genes (PLS1−). The dominant cluster reflects enrichment of Parkinson’s disease and neurodegeneration pathways, including the KEGG Parkinson disease pathway, aerobic respiration and respiratory electron transport, mitochondrion organization, mitochondrial protein degradation, glycolysis and gluconeogenesis, and cellular stress response. More details are reported in Supplementary Figure S5.

In Cluster 2, PLS1-positive genes were enriched in nuclear regulatory processes, including chromatin organization, DNA metabolic processes, and mRNA metabolism (Figure 4). Conversely, PLS1-negative genes showed strong enrichment in mitochondrial and bioenergetic pathways, including aerobic respiration, electron transport chain activity, and mitochondrial organization, as well as the Parkinson’s disease KEGG pathway (Figure 4). These findings suggest that regions exhibiting the greatest MIND reductions in Cluster 2 are associated with transcriptional signatures indicative of mitochondrial dysfunction and neurodegenerative processes.

### 2.5. Cell-Type Specificity

To investigate the cellular substrates underlying these transcriptional patterns, cell-type enrichment analysis was performed using a hypergeometric test across canonical cortical cell classes, with FDR correction. In Cluster 1, no significant enrichment was observed for PLS1-positive genes. In contrast, PLS1-negative genes showed significant enrichment for oligodendrocyte-specific markers (fold enrichment = 6.93, p-FDR < 0.001; Supplementary Figure S2), indicating a relative underrepresentation of oligodendrocyte-related transcriptional signatures in regions with increased MIND strength. No other cell types reached statistical significance.

In Cluster 2, neither PLS1-positive nor PLS1-negative gene sets showed significant cell-type enrichment after FDR correction. A nominal enrichment for excitatory neurons was observed among PLS1-negative genes (fold enrichment = 2.64, p-raw = 0.041), but this did not survive multiple comparison correction (p-FDR = 0.288).

### 2.6. Correlations Between Regional MIND Strength and Serum Biomarkers

To investigate the relationship between structural network alterations and both clinical measures and systemic markers of neurodegeneration, Spearman correlations were performed between regional MIND strength and clinical variables and serum biomarker levels, restricted to regions showing significant case–control differences (FWER-corrected; Supplementary Table S3). In Cluster 1, significant associations were observed between regional MIND strength and motor severity. Specifically, MIND strength in the left middle temporal cortex was positively correlated with both MDS-UPDRS total scores (r = 0.358, p-FDR = 0.006) and motor examination scores (MDS-UPDRS-III; r = 0.284, p-FDR = 0.030), indicating that increased structural similarity in this region is associated with greater motor impairment. No significant correlations were observed between MIND strength and serum biomarkers after FDR correction.

In Cluster 2, MIND strength showed significant associations with both clinical and serum measures. Regarding clinical variables, disease onset age was negatively correlated with MIND strength in bilateral anterior cingulate cortex (left and right rostral divisions) and orbitofrontal regions (medial and lateral), indicating that earlier disease onset is associated with reduced structural similarity in fronto-limbic regions. Stronger associations were observed with serum biomarkers. Ten significant negative correlations were identified across multiple cortical regions, predominantly involving fronto-cingulate and orbitofrontal cortices. Reduced MIND strength in these regions was associated with higher serum levels of neurofilament light chain (NfL; r range: −0.415 to −0.497) and glial fibrillary acidic protein (GFAP; r range: −0.459 to −0.496), consistent with increased axonal damage and astrocytic activation (Supplementary Table S3). Additional associations extended to inferior parietal and inferior frontal regions, including the pars orbitalis and pars triangularis.

These findings indicate that structural network disruption in Cluster 2 is closely linked to markers of axonal damage and astrocytic activation, suggesting a more pronounced neurodegenerative phenotype. Although serum NfL and GFAP concentrations did not differ significantly across groups (Table 2), the within-cluster correlations capture continuous interindividual covariation between biomarker levels and regional MIND strength. These findings indicate that lower regional MIND strength in Cluster 2 is associated with markers of axonal injury and astrocytic activation, consistent with a greater neurodegenerative burden.

## 3. Discussion

In this study, we examined how individual-level morphometric network alterations, cortical gene expression, and serum markers of neurodegeneration converge to define biologically distinct subtypes of Parkinson’s disease. No significant MIND differences emerged when patients were analysed as a single group. Rather, by modelling deviations from healthy controls within a discriminative framework, HYDRA recovered two robust subtypes with divergent MIND profiles [13,14,23]. These spatially distinct patterns may cancel each other out in conventional case–control analyses [24]. The two subtypes were only partially distinguished by clinical measures, differing in tremor scores and age at onset, whereas overall motor severity, disease duration, and Hoehn and Yahr stage were comparable. Importantly, they did not map onto the classical motor phenotypes: both tremor-dominant (TD) and postural instability/gait difficulty (PIGD) patients were represented in each subtype (Cluster 1: PIGD 58%, TD 42%; Cluster 2: PIGD 70%, TD 30%), and although this distribution differed between subtypes (χ^2^, *p* < 0.001), neither corresponded to a single phenotype. These findings suggest that morphometric stratification captures a dimension of disease heterogeneity that is only partially reflected by the conventional TD/PIGD dichotomy and may reflect underlying disease configurations that are not fully captured by clinical classifications alone.

In Cluster 1, MIND increases were concentrated within temporo-parietal and sensorimotor regions, predominantly involving granular and associative posterior cortices according to Yeo network and von Economo analyses. These alterations were spatially coupled with a posterior-to-anterior transcriptional gradient (PLS1, r = 0.589, p-spin < 0.001; 28.9% of variance). Regions showing the greatest MIND increase were enriched in biological processes related to synaptic organisation and plasticity, consistent with the molecular profile of synaptically active associative cortices [25,26]. The most prominent cell-type signal was a marked under-representation of oligodendrocyte-specific transcripts in these same regions (fold enrichment = 6.93, p-FDR < 0.001). Given the role of oligodendrocytes in supporting metabolically demanding axons through activity-dependent coupling [27], oligodendrocyte-related transcriptional signatures may render these cortical regions particularly vulnerable. Experimental evidence indicates that α-synuclein pathology can impair oligodendrocyte function [28], raising the possibility that oligodendroglial dysfunction may contribute to the regional susceptibility observed in this subtype. Consistently, PD-associated genes involved in proteostasis and vesicular trafficking were underexpressed in the most affected regions (Supplementary Table S2). The absence of significant associations with serum biomarkers, together with the younger age at onset and higher tremor scores, suggests that the cortical changes observed in Cluster 1 may occur in relative dissociation from measurable systemic neurodegeneration.

In contrast to Cluster 1, MIND strength in Cluster 2 was diffusely reduced across frontal, cingulate, parietal, and insular cortices, with predominant involvement of agranular prefrontal regions, in agreement with previous evidence of fronto-parietal vulnerability in Parkinson’s disease [29,30,31]. The preferential involvement of prefrontal, cingulate, and orbitofrontal cortices showed partial spatial correspondence with the advanced neocortical stages proposed in the Braak framework [5], although such correspondence should be interpreted cautiously given the cross-sectional nature of the present study. MIND alterations were spatially coupled with a limbic-to-prefrontal transcriptional gradient (PLS1, r = 0.404, p-spin = 0.003; 20.3% of variance), and the most disrupted regions were enriched for mitochondrial and bioenergetic pathways. Notably, the Parkinson’s disease KEGG pathway emerged as the top enriched term, consistent with the involvement of canonical mechanisms of PD neurodegeneration, including mitochondrial dysfunction, dopaminergic synapse alterations, and impaired protein degradation [32,33,34]. PD-associated genes identified in these regions similarly converged on mitochondrial, lysosomal, and proteasomal processes involved in α-synuclein handling and clearance [35] (Supplementary Table S2). In contrast to Cluster 1, no cell-type enrichment survived multiple-comparison correction, suggesting that the molecular signature of Cluster 2 is less cell-type specific and may reflect a broader metabolic vulnerability.

The strongest evidence supporting a greater neurodegenerative burden in Cluster 2 came from serum biomarkers. Reduced MIND strength within fronto-cingulate and orbitofrontal regions correlated with higher serum NfL (r = −0.415 to −0.497) and GFAP (r = −0.459 to −0.496), with the same cortical areas showing the most distinct neurodegenerative profile and the strongest enrichment for mitochondrial pathways. Earlier disease onset was independently associated with greater fronto-limbic disruption after adjustment for disease duration. The concomitant elevation of NfL, a marker of neuroaxonal injury [20,36], and GFAP, a marker of astrocytic activation [22,37], suggests that network disintegration in this subtype is accompanied by neuroaxonal damage and reactive gliosis.

Overall, the convergence of structural disruption, transcriptional signatures, and circulating biomarkers within the same fronto-cingulate circuitry points to a greater neurodegenerative burden in Cluster 2. However, serum NfL and GFAP did not differ significantly across HC, Cluster 1, and Cluster 2, although NfL showed a non-significant trend (*p* = 0.081). This does not conflict with the significant correlations observed within Cluster 2, as group comparisons assess differences in overall biomarker distributions, whereas correlation analyses test whether interindividual biomarker variation covaries with regional MIND strength. These findings therefore indicate continuous imaging–biomarker associations within Cluster 2 rather than serum biomarker-based separation of the subtypes.

These divergent patterns should be interpreted considering what MIND strength represents. Rather than measuring cortical atrophy directly, MIND indexes the degree of architectural similarity among cortical areas relative to the rest of the cortex [38]. In Cluster 2, the widespread MIND reduction therefore represents the network-level counterpart of the grey matter degeneration commonly reported in Parkinson’s disease, an interpretation reinforced by its spatial overlap with mitochondrial signatures and serum markers of injury. By contrast, the focal MIND increases observed in Cluster 1 should not be interpreted as evidence of preserved tissue integrity; rather, greater inter-regional similarity may reflect reduced architectural differentiation across neighbouring cortical regions. This pattern is not necessarily compensatory: increased MIND strength in the left middle temporal cortex correlated with greater motor severity, and the same regions showed a distinct oligodendroglial and synaptic molecular signature. These observations suggest that the two subtypes may reflect different modes of cortical involvement rather than successive stages of a single atrophic process. This view is further supported by the two PD-associated genes shared across subtypes, DNAJC13 and GBA2, which point to common proteostatic and vesicular mechanisms transcending subtype boundaries [39], and argues against interpreting Cluster 1 as merely an earlier stage of Cluster 2, although longitudinal studies are required to confirm this [4,40].

These findings suggest that morphometric stratification may provide an in vivo framework for identifying biologically distinct patterns of cortical involvement independently of clinical motor phenotype, with potential implications for patient stratification and disease monitoring.

### Limitations

Several limitations should be acknowledged. First, follow-up studies are needed to clarify whether the identified subtypes represent stable biological entities or different stages along a common disease trajectory.

Second, although MIND provides a powerful framework for mapping inter-regional structural similarity, its biological interpretation relies on two key assumptions: (1) that macrostructural MRI similarity reflects underlying cytoarchitectonic organization, and (2) that architectonically similar regions are more likely to be axonally connected, consistent with the homophily principle. While growing evidence supports these assumptions, they remain indirect, and MIND-based networks should therefore be interpreted as statistical proxies of cortical organization rather than direct measures of anatomical connectivity.

Third, the imaging–transcriptomic analysis is based on gene expression data from the Allen Human Brain Atlas, which comes from a small number of post-mortem healthy donors. In addition, the use of mirrored hemispheres and normative data may not fully capture the transcriptional landscape of Parkinson’s disease. For this reason, the associations we report should be interpreted as spatial correspondences rather than direct biological mechanisms.

Fourth, the current implementation of MIND focuses on the cortex and does not include subcortical or brainstem structures, which are known to play a key role in Parkinson’s disease. Although cortical changes may partly reflect downstream effects of subcortical pathology, integrating subcortical measures and multimodal connectivity data would provide a more complete picture of disease-related network alterations.

Fifth, the present analyses are cross-sectional, and the associations observed between MIND strength and serum biomarkers of neurodegeneration should therefore be interpreted as concurrent correspondences rather than evidence of an active or progressive degenerative process; longitudinal sampling will be required to establish their temporal dynamics.

Finally, the single-center design may limit the generalizability of the findings, which should therefore be confirmed in larger multi-site cohorts.

## 4. Materials and Methods

### 4.1. Subjects and Clinical Evaluation

This study enrolled 200 individuals with Parkinson’s disease and 121 neurologically healthy control participants. Patients with PD were consecutively recruited between 2020 and 2025 at the Institute of Neurology, University Magna Graecia of Catanzaro, Italy. PD diagnosis was established according to the most recent international clinical diagnostic criteria [1]. All patients with PD underwent an extensive neurological examination in the practically defined OFF medication state, following overnight withdrawal of dopaminergic treatment. Evaluations were performed by movement disorders specialists and included the Movement Disorder Society-sponsored revision of the Unified Parkinson’s Disease Rating Scale (MDS-UPDRS) and disease staging using the Hoehn and Yahr (H–Y) scale. Tremor and postural instability/gait difficulty were computed, and a tremor-to-PIGD ratio was derived. Patients with a ratio ≥ 1.15 were classified as tremor-dominant (TD), whereas those with a ratio ≤ 0.90 were classified as PIGD [2]. Exclusion criteria for PD patients included clinical features suggestive of alternative neurological diagnoses, normal striatal dopaminergic uptake on ^123^I-FP-CIT SPECT, and MRI evidence of other neurological conditions such as brain tumors, progressive supranuclear palsy, multiple system atrophy, normal pressure hydrocephalus, basal ganglia lacunar infarctions, or extensive subcortical vascular pathology. Healthy controls had no history of neurological, psychiatric, or major systemic disorders. The study was approved by the Regional Ethics Committee of Calabria, University Magna Graecia of Catanzaro, Italy (approval code 358/2020; approval date 10 November 2020), and all participants provided written informed consent in accordance with the Declaration of Helsinki.

### 4.2. MRI Acquisition and Image Preprocessing

Magnetic resonance imaging (MRI) was performed using either a 3.0 Tesla GE Discovery MR750 scanner equipped with an 8-channel head coil (GE Healthcare, Milwaukee, WI, USA) or a 3.0 Tesla Siemens Biograph mMR scanner (Siemens Healthineers, Forchheim, Germany), following previously described acquisition protocols [41,42]. Briefly, for the GE system, a three-dimensional T1-weighted volumetric spoiled gradient echo sequence was acquired in the sagittal plane (repetition time [TR] = 9.2 ms, echo time [TE] = 3.7 ms, slice thickness = 1.0 mm, matrix size = 256 × 256, flip angle = 12°, field of view [FOV] = 256 mm). For the Siemens system, a three-dimensional T1-weighted magnetization-prepared rapid acquisition gradient echo (MPRAGE) sequence was acquired (TR = 2300 ms, TE = 2.34 ms, voxel size = 1.0 × 1.0 × 1.0 mm^3^, FOV = 256 × 256 mm^2^). Scanner type was included as a covariate in downstream statistical analyses to account for inter-scanner variability. All scans were visually inspected for quality, and images with significant motion artifacts were excluded. T1-weighted images were processed using FreeSurfer (version 7.1; Massachusetts General Hospital, Harvard Medical School). Cortical reconstruction and volumetric segmentation were performed using the standard recon-all pipeline, including skull stripping, Talairach transformation, intensity normalization, segmentation of subcortical white and deep gray matter structures, reconstruction of the gray–white matter boundary, topological correction, and surface deformation to accurately identify the gray matter–white matter and gray matter–cerebrospinal fluid interfaces. Cortical parcellation was performed according to the Desikan–Killiany atlas, yielding 34 anatomically defined cortical regions per hemisphere.

Morphometric features were computed using both surface- and volume-based approaches, including cortical thickness, surface area, gray matter volume, sulcal depth, and mean curvature extracted at the vertex level [43] (Supplementary Figure S3). Quality control was further assessed using the Euler number [44], an automated metric provided by FreeSurfer. No scans showed Euler number values below −120, indicating that all reconstructions met established quality thresholds.

### 4.3. Construction of Subject-Specific MIND Networks

Structural similarity between cortical regions was quantified using the Morphometric INverse Divergence (MIND) framework, which applies a multivariate statistical approach based on Kullback–Leibler (KL) divergence. For each subject, vertex-wise morphometric features derived from the FreeSurfer pipeline (cortical thickness, surface area, gray matter volume, sulcal depth, and mean curvature) were extracted and retained at the vertex level within each Desikan–Killiany atlas region. Thus, each cortical region was represented as a multivariate distribution of morphometric features rather than a single summary value. For each pair of regions, MIND quantified the divergence between these multivariate distributions using a k-nearest neighbor-based approximation of KL divergence, avoiding explicit probability density estimation and reducing sensitivity to parameter selection [9]. MIND values were bounded between 0 and 1, with higher values indicating greater structural similarity between regions. Vertices with biologically implausible values (e.g., zero thickness, volume, or sulcal depth) were excluded prior to analysis. For each participant, these procedures yielded a symmetric 68 × 68 subject-specific MIND connectivity matrix (Supplementary Figure S3). Additionally, regional MIND strength was computed by averaging all the connections between one region and the other 67 cortical regions in both cohorts.

To account for potential scanner-related variability, harmonization of MIND strength values was performed using the neuroComBat method (https://github.com/Jfortin1/neuroCombat, accessed on 10 January 2026). This step was necessary because MRI data were acquired using two different 3.0 Tesla scanners, which may introduce systematic non-biological differences related to hardware and acquisition protocols. Scanner type was modeled as the batch variable.

### 4.4. PD Subtyping Using HYDRA

Subgroup identification within the PD cohort was performed using the HYDRA framework, a semi-supervised machine learning approach which delineates disease heterogeneity by directly contrasting individuals with PD against HC participants [14]. Unlike conventional supervised classification methods, such as support vector machines or random forest algorithms, which focus solely on diagnostic discrimination and do not resolve within-group heterogeneity, HYDRA integrates discriminative modeling with unsupervised clustering to uncover latent subtypes within the patient population. Specifically, the algorithm constructs multiple linear maximum-margin hyperplanes to separate healthy controls from PD patients, thereby defining a multi-dimensional discriminative space. Patient subgroups are subsequently identified based on their alignment with distinct facets of this geometric structure, represented by individual hyperplanes. The HYDRA was performed using regional MIND strength values as input features with the following parameters: 50 alternating optimization cycles between hyperplane learning and cluster assignment, 20 consensus clustering iterations, a regularization coefficient set to 0.25, and a 10-fold cross-validation scheme [13]. The robustness and reproducibility of the derived clusters were evaluated using the Adjusted Rand Index (ARI), which quantifies agreement between clustering solutions obtained across cross-validation folds while correcting for chance-level overlap [45].

### 4.5. Case–Control Analysis of MIND Measures Across PD Subtypes

To examine differences in regional MIND strength between PD subtypes and healthy control participants, linear regression analyses were conducted for each cortical region. Regional MIND values were entered as dependent variables, while age, sex and total intracranial volume (TIV) were included as covariates:

MIND*_i_* = β_0_ + β_1_ × Age + β_2_ × Sex + β_3_ × TIV + β_4_ × Group + ε

where MIND_i_ represents the MIND strength of region i and Group is a categorical variable coding PD subtype versus HC. Residualized MIND values (after covariate adjustment) were further organized according to two established neuroanatomical classification frameworks. Functional network affiliation was defined using the seven-network Yeo parcellation based on resting-state functional connectivity [46], while cytoarchitectonic categorization was derived from the von Economo atlas [47,48]. Within each functional network and cytoarchitectonic class, mean residualized MIND strength was computed by averaging values across constituent regions.

### 4.6. Transcriptomic Data

Regional gene expression data were obtained from the Allen Human Brain Atlas (AHBA), which provides genome-wide microarray expression profiles from six postmortem adult human brains sampled across thousands of spatial locations (http://human.brain-map.org) [49]. Gene expression data were preprocessed using the Abagen toolbox, following established imaging–transcriptomics pipelines (https://github.com/rmarkello/abagen, accessed on 12 January 2026) [50]. Briefly, microarray probes were reannotated to gene symbols, and probes with low expression intensity were excluded. For genes represented by multiple probes, the probe with the highest regional stability across donors was retained. Tissue samples were then assigned to brain regions based on their spatial proximity. Gene expression values were normalized across samples using a scaled robust sigmoid transformation to reduce inter-sample variability. To enable whole-brain analyses, gene expression profiles were mapped onto the cortical parcellation used in this study, and values from the left hemisphere were mirrored to the right hemisphere, following previously established procedures [23]. This resulted in a region-by-gene expression matrix (68 parcels × 15,633 genes) used for subsequent imaging–transcriptomic analyses.

### 4.7. Imaging–Transcriptomic Association (PLS Regression)

To identify molecular correlates of spatial patterns of cortical MIND alterations, partial least squares (PLS) regression was applied to relate regional gene expression profiles to imaging-derived phenotypes [51]. For each PD subtype, regional case–control t-statistic maps of MIND strength were used as the response vector, while the standardized expression levels of all genes across cortical regions served as predictors. Both imaging and gene expression data were z-score normalized prior to analysis. PLS components were derived using singular value decomposition, identifying orthogonal latent variables that maximize the covariance between gene expression and neuroimaging data. The first latent component (PLS1) represents a weighted linear combination of gene expression values whose spatial distribution across the cortex best corresponds to the observed pattern of MIND alterations.

To assess statistical significance, a spatial autocorrelation-preserving permutation test (spin test; 10,000 rotations) was performed [52]. Cortical gene expression maps were randomly rotated on the spherical surface while the t-statistic map was held fixed, preserving spatial covariance and generating a null distribution of R^2^. The spin-based *p*-value (p-spin) was computed by comparing the empirical R^2^ of PLS1 to this null distribution. To evaluate the robustness of gene contributions, bootstrap resampling was performed by sampling cortical regions with replacement (5000 iterations). For each gene, a Z-score was computed as the ratio between its PLS1 weight and the corresponding bootstrap-estimated standard error [53]. Genes were ranked according to their contribution to the imaging–transcriptomic association, and those with |Z| > 3 were retained following false discovery rate (FDR) correction. Regional PLS1 scores were projected back onto the cortical surface and correlated with the empirical MIND t-statistic maps to quantify the spatial correspondence between transcriptomic and neuroimaging patterns.

### 4.8. Transcriptomic Enrichment Analyses

Functional enrichment analysis of PLS1-weighted genes was performed to characterize the biological processes associated with MIND alterations. Genes were ranked according to their PLS1 weights and stratified into positively (PLS1+) and negatively (PLS1−) associated gene sets based on their Z-scores. Only genes exceeding the significance threshold (|Z| > 3, FDR-corrected) were retained for analyses.

Functional annotation was conducted using Metascape (https://metascape.org/gp/index.html, accessed on 13 January 2026), integrating multiple ontology sources, including Gene Ontology (GO) biological processes and Kyoto Encyclopedia of Genes and Genomes (KEGG) pathways. Enrichment significance was assessed using false discovery rate (FDR) correction, with a threshold of q < 0.05.

Next, to investigate the cellular substrates underlying these transcriptional patterns, cell-type enrichment analysis was performed by comparing PLS1 gene sets with reference gene signatures derived from single-cell transcriptomic datasets. Genes were classified into canonical brain cell types, including excitatory neurons, inhibitory neurons, astrocytes, microglia, oligodendrocytes, oligodendrocyte precursor cells (OPCs), and endothelial cells [18]. Overlap between PLS1+ or PLS1− gene sets and cell-type-specific gene lists was quantified. Statistical significance of overlap between PLS1 gene sets and cell-type-specific gene lists was assessed using the hypergeometric test, with the union of all genes tested across comparisons as background. Resulting *p*-values were corrected for multiple comparisons using FDR correction (Benjamini–Hochberg; q < 0.05).

Finally, to assess the disease relevance of these transcriptomic findings, PD-related genes were retrieved from the Allen Human Brain Atlas by querying the “Gene Classifications” database using the term “Parkinson disease”. The resulting gene set was filtered to retain only genes present in the background expression matrix used in the present study, ensuring consistency with the imaging–transcriptomic analysis. Overlap between PD-related genes and PLS1-ranked genes was then computed to identify disease-relevant transcriptional signatures associated with regional MIND alterations. For each overlapping gene, Spearman correlation analysis was performed between its regional expression profile and the corresponding case–control t-statistic map of MIND strength. Statistical significance was assessed using false discovery rate (FDR) correction, with a threshold of q < 0.05.

### 4.9. Serum Collection and Assessment of Biomarkers

Serum samples from each subject were collected in BD Vacutainer^TM^ SST^TM^ Serum Separation Tubes (BD, Franklin Lakes, NJ, USA) between 9 a.m. and 12 p.m. and processed within 30 min by centrifugation at 3000 rpm for 10 min at 4 °C, followed by aliquoting and storage at −80 °C until use. For biomarker evaluation, serum aliquots were thawed overnight at 4 °C, thoroughly mixed and centrifuged for 15 min at 2200 rpm. All the biomarkers were assessed by ultrasensitive single molecule array (SIMOA) technology on a fully automated Quanterix HD-X^TM^ Automated Immunoassay Analyzer (Quanterix, Billerica, MA, USA). In detail, NfL, GFAP, Aβ40 and Aβ42 were collectively measured using the Neurology 4-Plex E Advantage PLUS kit (104465, Quanterix), while p-tau217 was separately evaluated using the p-Tau 217 Advantage PLUS kit (104588, Quanterix). In both cases, manufacturer’s instructions to assess EDTA plasma samples were applied. All measurements were performed in duplicates and sample analysis was performed blindly, without prior knowledge of the patients’ diagnoses [54].

### 4.10. Statistical Analysis

All statistical analyses were conducted using RStudio 4.3.1. Variables were assessed for normality prior to statistical testing. Group comparisons of MIND measures were performed on residualized values after covariate adjustment.

PD clusters (k = 2) identified through the HYDRA framework were compared, and group differences in continuous demographic and cognitive variables (age, education, cognitive test scores) were evaluated using the Kruskal–Wallis test, followed by Bonferroni-corrected post hoc comparisons. Sex and motor phenotype distribution differences were assessed using the χ^2^ test. Clinical variables (age at onset, disease duration, LEDD, MDS-UPDRS-III scores, H–Y stage, tremor score) were compared between clusters using Mann–Whitney U tests or independent-samples *t*-tests, depending on data distribution. Group differences in serum biomarker levels (NfL, GFAP, Aβ42/Aβ40 ratio, and p-tau217) across HC, Cluster 1, and Cluster 2 were assessed using the Kruskal–Wallis test.

Following MIND strength estimation, group differences at the regional level were assessed using two-tailed Welch’s *t*-tests. To control for multiple comparisons across regions, a permutation-based family-wise error rate (FWER) correction was applied using 1000 permutations. Regions surviving FWER correction at *p* < 0.05 were considered statistically significant. For Yeo network- and von Economo class-based analyses, group differences were evaluated using Welch’s *t*-tests, with permutation-based FWER correction applied across network or cytoarchitectonic labels.

The associations between individual PLS1 scores and serum biomarkers (NfL, GFAP, Aβ42/Aβ40 ratio, and p-tau217) were examined separately within each cluster. Correlation analyses were performed using Spearman’s correlation coefficient. Resulting *p*-values were corrected for multiple comparisons within each group using the Benjamini–Hochberg false discovery rate (FDR) procedure (q < 0.05).

## 5. Conclusions

In conclusion, individual-level morphometric stratification reveals that Parkinson’s disease encompasses biologically distinct subtypes that are masked in group-level analyses and only partially captured by conventional motor phenotypes. Integrating HYDRA-derived MIND subtypes with transcriptomic and serum biomarkers identified two distinct cortical patterns: posterior cortical reorganization associated with synaptic–oligodendroglial signatures and no significant serum biomarker associations, and fronto-limbic MIND reductions associated with mitochondrial pathways and continuous associations with NfL and GFAP. These findings suggest that patients with comparable motor presentations may have distinct biological profiles. By resolving this heterogeneity, the proposed multimodal framework provides a potential basis for moving beyond symptom-based classification toward biologically informed patient stratification. If validated longitudinally and in independent multisite cohorts, such subtype-specific characterization could support clinical trial enrichment, biomarker-guided patient selection for disease-modifying therapies, and individualized monitoring, thereby contributing to precision medicine approaches in Parkinson’s disease.

## Figures and Tables

**Table 1:** Demographic and clinical data of Parkinson’s disease and control subjects.

Variable	HC (n = 121)	PD (n = 200)	*p*-Value
**Demographics**			
Sex (0/1)	57 (47%)/64 (53%)	74 (37%)/126 (63%)	0.0952 ^a^
Age (years) ^b^	65.69 ± 8.14	66.70 ± 9.95	0.1361 ^c^
**Clinical**			
Tremor score ^b^	—	0.35 ± 0.38	—
MDS-UPDRS motor ^b^	—	22.15 ± 14.37	—
MDS-UPDRS total ^b^	—	39.56 ± 21.53	—
H–Y stage ^b^	—	1.63 ± 0.72	—
Disease duration (years) ^b^	—	4.85 ± 4.40	—
Disease onset (years) ^b^	—	61.77 ± 10.15	—
LEDD (mg) ^b^	—	362.73 ± 337.32	—
**Serum biomarkers**			
Aβ42/Aβ40 ratio ^b^	0.13 ± 0.02	0.14 ± 0.09	0.3498 ^d^
p-tau217 (pg/mL) ^b^	0.18 ± 0.23	0.19 ± 0.21	0.1627 ^d^
GFAP (pg/mL) ^b^	75.49 ± 31.04	82.48 ± 51.80	0.9107 ^d^
NfL (pg/mL) ^b^	10.90 ± 7.39	13.15 ± 9.67	0.2235 ^d^
Aβ40 (pg/mL) ^b^	22.17 ± 14.26	22.99 ± 17.94	0.8351 ^d^
Aβ42 (pg/mL) ^b^	3.32 ± 2.01	2.88 ± 1.66	0.3688 ^d^

Abbreviations: PD = Parkinson’s disease; HC = healthy controls; LEDD = levodopa equivalent daily dose; MDS-UPDRS = Movement Disorder Society–Unified Parkinson’s Disease Rating Scale; H–Y = Hoehn and Yahr. ^a^ Fisher’s exact test (χ^2^). ^b^ Data are expressed as mean ± standard deviation. ^c^ *t*-test. ^d^ Mann–Whitney U test.

**Table 2:** Demographic and clinical data of PD clusters and control subjects.

Variable	HC (n = 121)	Cluster 1 (n = 96)	Cluster 2 (n = 104)	*p*-Value
**Demographics**				
Sex (0/1)	57 (47%)/64 (53%)	23 (24%)/73 (76%)	51 (49%)/53 (51%)	**0.0003** ^a^
Age (years) ^b^	65.69 ± 8.14	64.56 ± 10.10 #	68.68 ± 9.44 *	**0.0046** ^c^
**Clinical**				
Tremor score ^b^	—	0.41 ± 0.40	0.29 ± 0.35	**0.0259** ^d^
MDS-UPDRS motor ^b^	—	21.95 ± 14.47	22.34 ± 14.34	0.8920 ^d^
MDS-UPDRS total ^b^	—	38.46 ± 20.94	40.56 ± 22.13	0.6286 ^d^
H–Y stage ^b^	—	1.54 ± 0.64	1.72 ± 0.78	0.1299 ^e^
Disease duration (years) ^b^	—	4.43 ± 4.08	5.24 ± 4.66	0.3060 ^e^
Disease onset (years) ^b^	—	60.15 ± 10.44	63.26 ± 9.69	**0.0298** ^d^
LEDD (mg) ^b^	—	350.34 ± 361.12	373.74 ± 316.30	0.4613 ^d^
**Serum biomarkers**				
Aβ42/Aβ40 ratio ^b^	0.13 ± 0.02	0.13 ± 0.07	0.14 ± 0.10	0.5600 ^c^
p-tau217 (pg/mL) ^b^	0.18 ± 0.23	0.23 ± 0.29	0.16 ± 0.09	0.3748 ^c^
GFAP (pg/mL) ^b^	75.49 ± 31.04	76.29 ± 43.35	88.22 ± 58.51	0.5655 ^c^
NfL (pg/mL) ^b^	10.90 ± 7.39	11.98 ± 9.02	14.23 ± 10.22	0.0807 ^c^

Abbreviations: PD = Parkinson’s disease; HC = healthy controls; LEDD = levodopa equivalent daily dose; MDS-UPDRS = Movement Disorder Society–Unified Parkinson’s Disease Rating Scale; H–Y = Hoehn and Yahr. ^a^ Fisher’s exact test. ^b^ Data are expressed as mean ± standard deviation. ^c^ Kruskal–Wallis rank test, followed by Bonferroni correction. ^d^ Student’s *t*-test. ^e^ Mann–Whitney U test. Significant *p*-values are in bold. * Cluster 2 > HC; # Cluster 1 < Cluster 2.

## Data Availability

The data presented in this study are available on request from the corresponding author. The data are not publicly available due to privacy and ethical restrictions. Gene expression data are publicly available from the Allen Human Brain Atlas (http://human.brain-map.org).

## Supplementary Materials

The following supporting information can be downloaded at: https://www.mdpi.com/article/10.3390/ijms27156769/s1. Reference [18] is cited in the supplementary materials.
