# Supplementary material for: Morphometric Inverse Divergence Networks Combined with HYDRA Identify Parkinson’s Disease Subtypes with Distinct Transcriptomic and Serum Biomarker Profiles
Source: Int J Mol Sci. 2026 Jul 28;27(15):6769. doi: 10.3390/ijms27156769 (PMC13466820; doi:10.3390/ijms27156769)
Supplement: Supplementary file 1 [file ijms-27-06769-s001.zip › ijms-4415965-supplementary.pdf]

Supplementary Figure S1. Analysis of MIND strength differences across functional and cytoarchitectonic networks in PD subtypes.

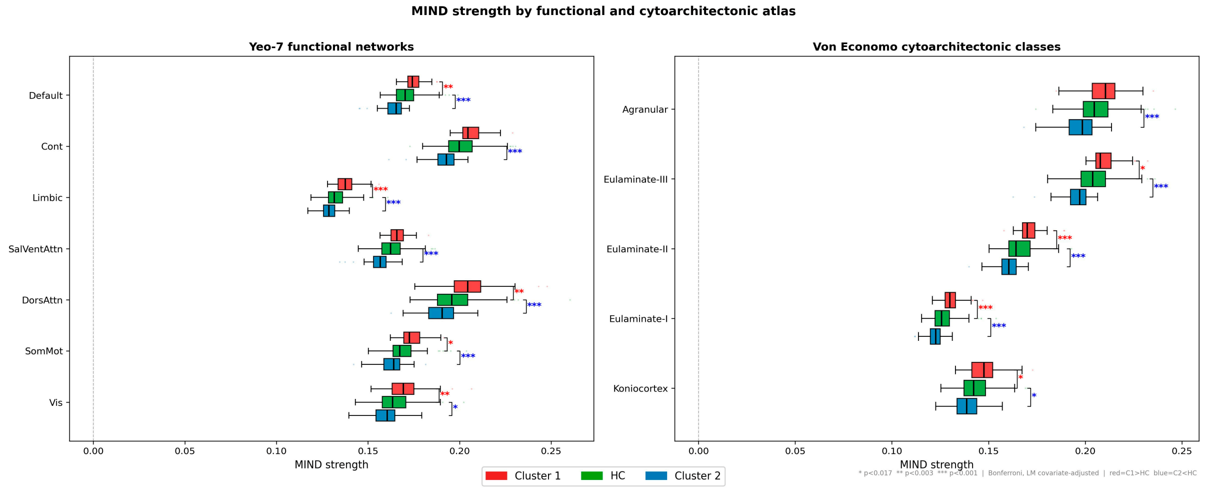

Left side. Yeo-7 functional network parcellation: group differences in regional MIND weighted strength across seven canonical functional networks (Visual, Somatomotor [SomMot], Dorsal Attention [DorsAttn], Salience/Ventral Attention [SalVentAttn], Limbic, Control [Cont], and Default Mode).

Right side.Von Economo cytoarchitectonic parcellation: group differences in MIND strength across five cytoarchitectonic classes (Koniocortex, Eulaminate-I, Eulaminate-II, Eulaminate-III, and Agranular cortex). Horizontal bars represent group medians with interquartile ranges. Red asterisks denote significant differences between Cluster 1 and HC (C1 > HC); blue asterisks denote significant differences between Cluster 2 and HC (C2 < HC). Statistical significance was assessed using linear models covariate-adjusted for age, sex, and total intracranial volume, with Bonferroni correction for multiple comparisons (* $p < 0.017$, ** $p < 0.005$, *** $p < 0.001$). HC, healthy controls.

Supplementary Figure S2. Cell-type specificity of PLS1-significant genes in PD subtypes.

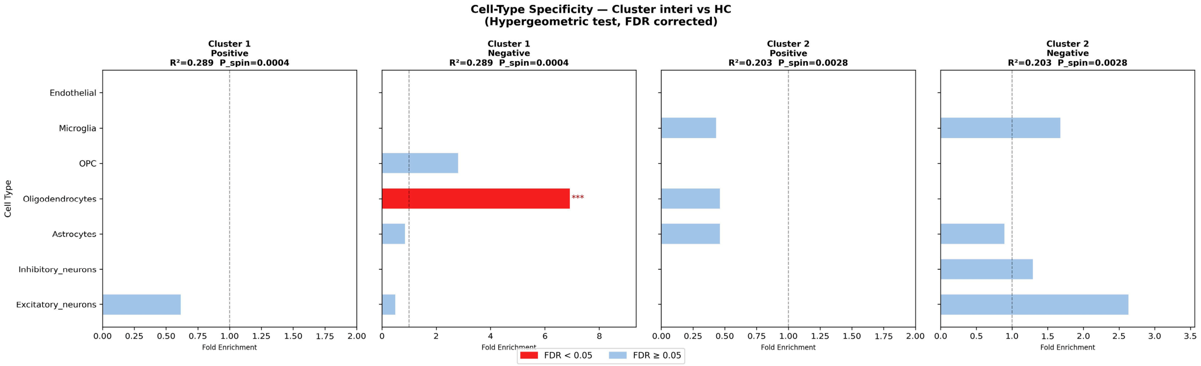

Bar plots display the fold enrichment of PLS1 positively and negatively weighted gene sets against seven canonical cortical cell-type reference gene lists (excitatory neurons, inhibitory neurons, astrocytes, oligodendrocytes, oligodendrocyte precursor cells [OPC], microglia, and endothelial cells), derived from human cortical single-cell RNA sequencing datasets (Wang et al.2024) Enrichment significance was assessed using the hypergeometric test with Benjamini-Hochberg FDR correction. Red bars indicate FDR < 0.05; blue bars indicate FDR ≥ 0.05. The dashed vertical line marks fold enrichment = 1 (no enrichment above chance). R^2 and P_spin values reported in each panel header refer to the PLS1 spatial correlation with the case-control t-map (spin permutation test, 10,000 rotations).
Cluster 1 negative genes (PLS1−) showed significant enrichment for oligodendrocytes (fold enrichment ~7, FDR < 0.001). No significant cell-type enrichment was observed for the remaining gene sets. HC, healthy controls; OPC, oligodendrocyte precursor cells; FDR, false discovery rate; PLS, partial least squares.

Supplementary Figure S3. Study workflow.

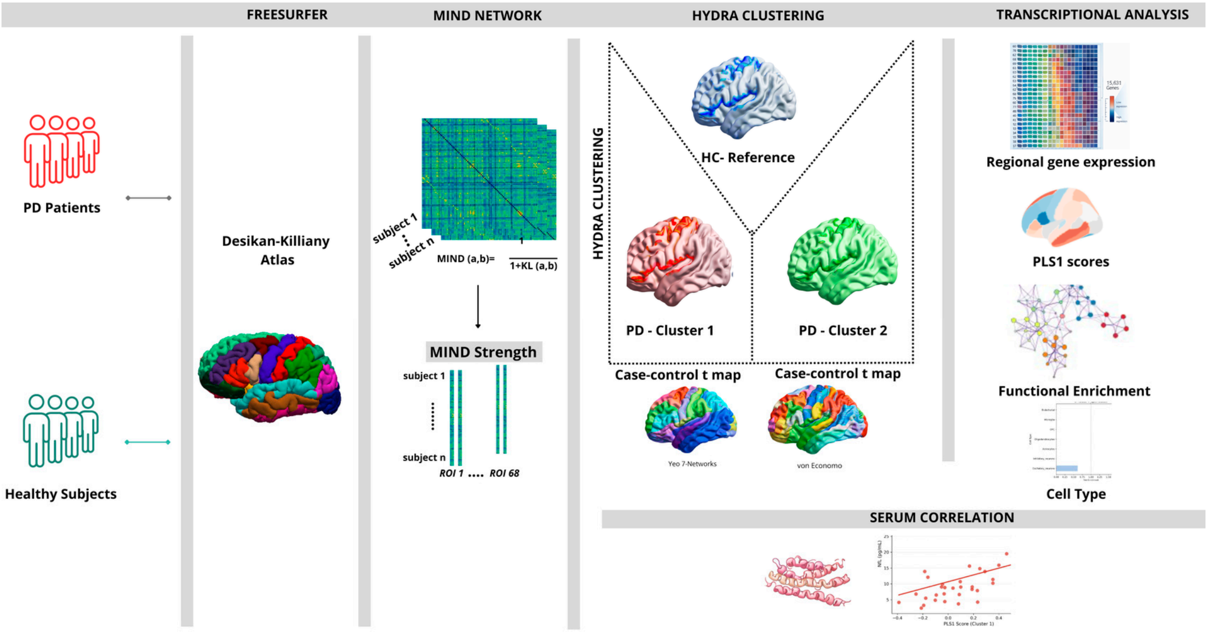

Individual-level Morphometric Inverse Divergence (MIND) networks were constructed from T1-weighted MRI data preprocessed with FreeSurfer (recon-all pipeline) for both Parkinson's disease (PD) patients and healthy controls (HC). Cortical surfaces were parcellated into 68 regions using the Desikan–Killiany (DK) atlas. For each subject, five macrostructural features (cortical thickness, surface area, gray matter volume, mean curvature, and sulcal depth) were extracted and combined to compute pairwise morphometric similarity using the Kullback–Leibler divergence-based MIND formula: MIND(a,b) = 1/(1+KL(a,b)), yielding a symmetric 68 × 68 similarity matrix per subject. Regional MIND strength was calculated as the average connectivity of each node across all 67 remaining regions, resulting in a subjects × 68 ROI feature matrix. The Heterogeneity through Discriminant Analysis (HYDRA) algorithm was applied to this feature matrix to identify distinct PD neuroanatomical subtypes relative to a healthy control reference, yielding two clusters: Cluster 1 (MIND strength increased vs HC) and Cluster 2 (MIND strength decreased vs HC). For each cluster, case–control t-maps of regional MIND strength differences were computed and visualized on cortical surfaces, and further summarized within Yeo 7 functional networks and von Economo cytoarchitectonic atlas. To identify transcriptomic correlates, regional gene expression profiles from the Allen Human Brain Atlas (AHBA; 68 regions × 15,633 genes) were linked to the case–control t-maps via partial least squares (PLS1) regression. PLS1 scores were projected onto individual subjects to yield individual transcriptomic alignment scores. Functional enrichment (Metascape) and cell-type specificity analyses were performed on PLS1-significant genes (|Z| > 3). Finally, regional MIND strength was correlated with serum biomarkers (NfL, GFAP, p-tau217, Aβ42/Aβ40 ratio) to integrate imaging-transcriptomic findings with peripheral markers of neurodegeneration. Abbreviations: PD, Parkinson's disease; HC, healthy controls; MIND, morphometric inverse divergence; HYDRA, heterogeneity through discriminant analysis; PLS, partial least squares; AHBA, Allen Human Brain Atlas; NfL, neurofilament light chain; GFAP, glial fibrillary acidic protein; ROI, region of interest.

Supplementary Figure S4. Enlarged, high-resolution version of the Cluster 1 enrichment networks (Figure 3B–C).

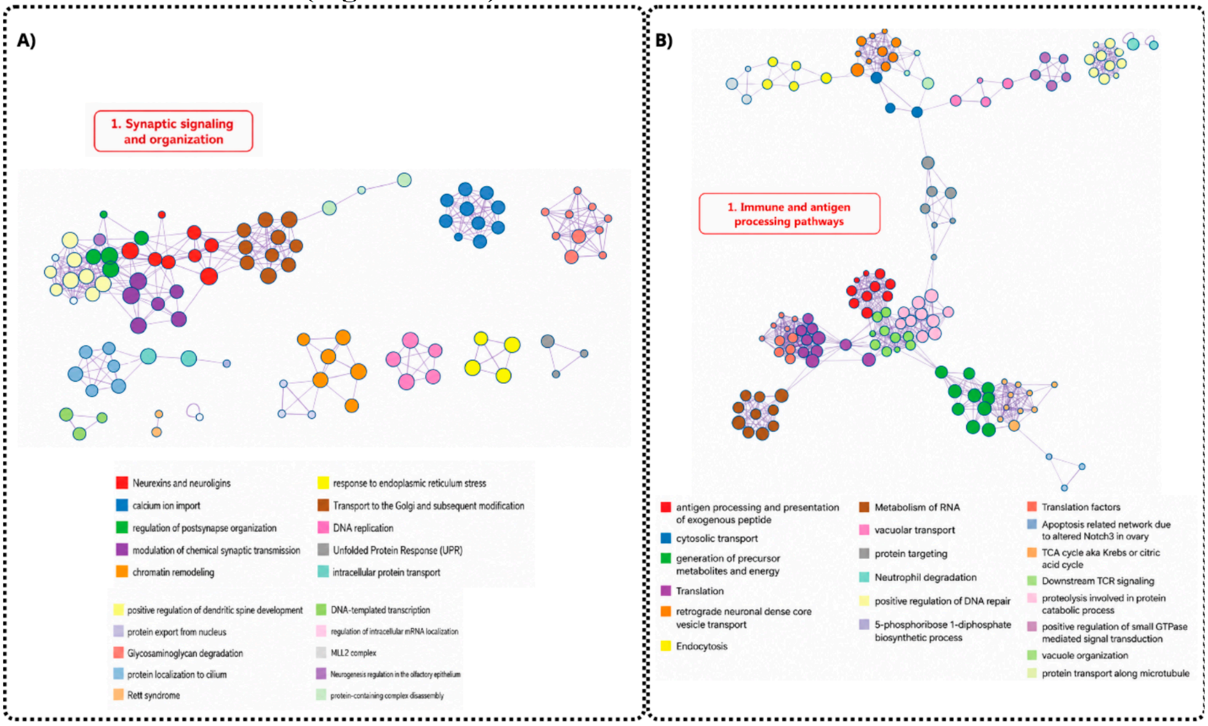

(A) Network plot for Cluster 1 positive genes (PLS1+). Each node represents an enriched biological term; edges connect terms sharing pathway membership above a kappa similarity threshold of 0.3. Node color denotes cluster identity; node size reflects gene count per term. The dominant cluster reflects convergent enrichment of synaptic signaling and organization pathways, including neurexin–neuroligin interactions, regulation of postsynapse organization, modulation of chemical synaptic transmission, and chromatin remodeling. (B) Network plot for Cluster 1 negative genes (PLS1−). The dominant cluster reflects enrichment of immune and antigen processing pathways, including antigen processing and presentation via MHC class II, cytosolic transport, generation of precursor metabolites and energy, translational regulation, and the TCA cycle.

Supplementary Figure S5 Enlarged, high-resolution version of the Cluster 2 enrichment networks (Figure 4B–C).

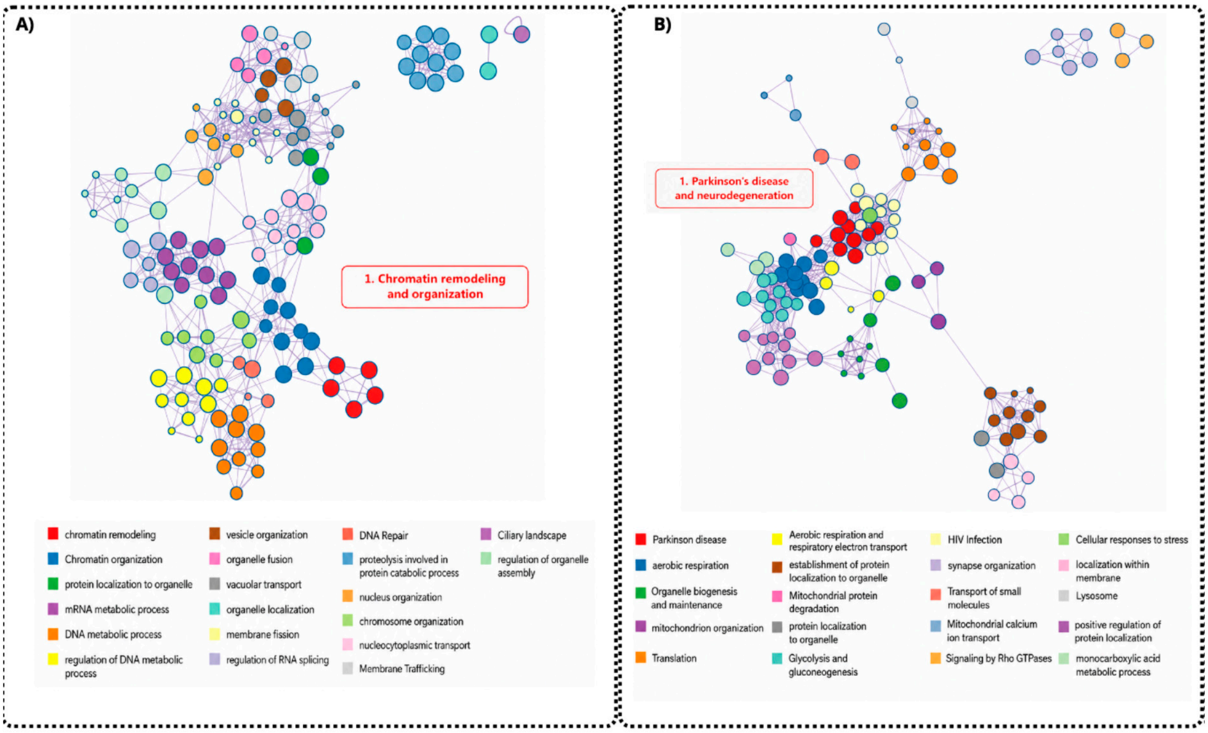

(A) Network plot for Cluster 2 positive genes (PLS1+). Each node represents an enriched biological term; edges connect terms sharing pathway membership above a kappa similarity threshold of 0.3. Node color denotes cluster identity; node size reflects gene count per term. The dominant cluster reflects convergent enrichment of chromatin remodeling and organization pathways, alongside DNA and mRNA metabolic processes, vesicle organization, organelle fusion, and nucleocytoplasmic transport. (B) Network plot for Cluster 2 negative genes (PLS1−). The dominant cluster reflects enrichment of Parkinson's disease and neurodegeneration pathways, including the KEGG Parkinson disease pathway, aerobic respiration and respiratory electron transport, mitochondrion organization, mitochondrial protein degradation, glycolysis and gluconeogenesis, and cellular stress response.

Supplementary Table S1. MIND strength data of PD Clusters versus control subjects.

ROI	ROI name	HC mean ± SD	Cluster 1 mean ± SD	t statistic	p_FWER
ROI_14	lh middletemporal	0.1951 ± 0.0133 (n=121)	0.2036 ± 0.0100 (n=96)	4.561	0.0020
ROI_28	lh superiorparietal	0.1977 ± 0.0144 (n=121)	0.2071 ± 0.0137 (n=96)	4.185	0.0050
ROI_21	lh postcentral	0.1805 ± 0.0154 (n=121)	0.1900 ± 0.0135 (n=96)	3.959	0.0120
ROI_63	rh superiortemporal	0.1915 ± 0.0121 (n=121)	0.1988 ± 0.0109 (n=96)	3.755	0.0210
ROI_48	rh middletemporal	0.1989 ± 0.0130 (n=121)	0.2058 ± 0.0102 (n=96)	3.618	0.0280
ROI_52	rh parsorbitalis	0.1493 ± 0.0113 (n=121)	0.1555 ± 0.0085 (n=96)	3.553	0.0340
**ROI**	**ROI name**	**HC mean ± SD**	**Cluster 2 mean ± SD**	**t statistic**	**p_FWER**
ROI_34	lh insula	0.1231 ± 0.0103 (n=121)	0.1167 ± 0.0073 (n=104)	-4.937	0.0010
ROI_41	rh inferiorparietal	0.2269 ± 0.0113 (n=121)	0.2155 ± 0.0089 (n=104)	-7.372	0.0010
ROI_64	rh supramarginal	0.2076 ± 0.0121 (n=121)	0.1990 ± 0.0083 (n=104)	-5.073	0.0010
ROI_27	lh superiorfrontal	0.2150 ± 0.0127 (n=121)	0.2036 ± 0.0103 (n=104)	-6.295	0.0010
ROI_61	rh superiorfrontal	0.2163 ± 0.0134 (n=121)	0.2047 ± 0.0100 (n=104)	-6.301	0.0010
ROI_7	lh inferiorparietal	0.2215 ± 0.0120 (n=121)	0.2103 ± 0.0107 (n=104)	-6.234	0.0010
ROI_60	rh rostralmiddlefrontal	0.2156 ± 0.0131 (n=121)	0.2059 ± 0.0104 (n=104)	-4.808	0.0010
ROI_59	rh rostralanteriorcingulate	0.1302 ± 0.0114 (n=121)	0.1200 ± 0.0111 (n=104)	-5.569	0.0010
ROI_24	lh precuneus	0.2146 ± 0.0116 (n=121)	0.2039 ± 0.0096 (n=104)	-6.396	0.0010
ROI_23	lh precentral	0.2071 ± 0.0116 (n=121)	0.1985 ± 0.0096 (n=104)	-5.184	0.0010
ROI_58	rh precuneus	0.2164 ± 0.0118 (n=121)	0.2064 ± 0.0101 (n=104)	-5.720	0.0010
ROI_45	rh lateralorbitofrontal	0.1858 ± 0.0140 (n=121)	0.1758 ± 0.0111 (n=104)	-4.599	0.0020
ROI_18	lh parsorbitalis	0.1468 ± 0.0106 (n=121)	0.1389 ± 0.0102 (n=104)	-4.524	0.0020
ROI_53	rh parstriangularis	0.1928 ± 0.0112 (n=121)	0.1850 ± 0.0102 (n=104)	-4.434	0.0020
ROI_36	rh caudalanteriorcingulate	0.1231 ± 0.0086 (n=121)	0.1175 ± 0.0077 (n=104)	-4.262	0.0030
ROI_25	lh rostralanteriorcingulate	0.1265 ± 0.0118 (n=121)	0.1179 ± 0.0106 (n=104)	-4.365	0.0030
ROI_2	lh caudalanteriorcingulate	0.1255 ± 0.0086 (n=121)	0.1193 ± 0.0084 (n=104)	-4.297	0.0030
ROI_11	lh lateralorbitofrontal	0.1849 ± 0.0138 (n=121)	0.1750 ± 0.0139 (n=104)	-4.195	0.0030
ROI_26	lh rostralmiddlefrontal	0.2211 ± 0.0132 (n=121)	0.2120 ± 0.0113 (n=104)	-4.219	0.0030
ROI_17	lh parsopercularis	0.1983 ± 0.0112 (n=121)	0.1897 ± 0.0143 (n=104)	-4.070	0.0070
ROI_62	rh superiorparietal	0.1967 ± 0.0148 (n=121)	0.1883 ± 0.0110 (n=104)	-4.060	0.0070
ROI_57	rh precentral	0.2037 ± 0.0126 (n=121)	0.1967 ± 0.0090 (n=104)	-3.982	0.0100
ROI_13	lh medialorbitofrontal	0.1775 ± 0.0115 (n=121)	0.1696 ± 0.0114 (n=104)	-3.972	0.0100
ROI_51	rh parsopercularis	0.1896 ± 0.0124 (n=121)	0.1822 ± 0.0113 (n=104)	-3.732	0.0130
ROI_56	rh posteriorcingulate	0.1485 ± 0.0112 (n=121)	0.1416 ± 0.0104 (n=104)	-3.749	0.0130
ROI_22	lh posteriorcingulate	0.1510 ± 0.0120 (n=121)	0.1435 ± 0.0109 (n=104)	-3.711	0.0150
ROI_37	rh caudalmiddlefrontal	0.1990 ± 0.0141 (n=121)	0.1909 ± 0.0108 (n=104)	-3.621	0.0190
ROI_10	lh lateraloccipital	0.1899 ± 0.0158 (n=121)	0.1820 ± 0.0126 (n=104)	-3.608	0.0210
ROI_30	lh supramarginal	0.2172 ± 0.0136 (n=121)	0.2093 ± 0.0112 (n=104)	-3.527	0.0310
ROI_19	lh parstriangularis	0.1833 ± 0.0102 (n=121)	0.1766 ± 0.0113 (n=104)	-3.488	0.0330
ROI_47	rh medialorbitofrontal	0.1812 ± 0.0117 (n=121)	0.1739 ± 0.0107 (n=104)	-3.435	0.0390
ROI_3	lh caudalmiddlefrontal	0.1986 ± 0.0137 (n=121)	0.1912 ± 0.0105 (n=104)	-3.381	0.0460

Supplementary Table S2. Spatial correlation between regional expression of PD-classified genes and case-control MIND strength t-maps in Cluster 1 and Cluster 2.

Cluster	Gene	Direction	Z-score	Spearman r	p_FDR
Cluster 1	VPS26A	negative	-4.447	-0.362	0.0143
Cluster 1	PSMA4	negative	-3.705	-0.291	0.0349
Cluster 1	**GBA2**	negative	-3.090	-0.266	0.0349
Cluster 1	CYCS	negative	-3.975	-0.265	0.0349
Cluster 1	**DNAJC13**	positive	3.752	0.281	0.0349
Cluster 2	PSMA7	negative	-3.270	-0.309	0.0333
Cluster 2	**GBA2**	negative	-3.773	-0.299	0.0341
Cluster 2	STX3	negative	-4.044	-0.269	0.0435
Cluster 2	HTRA2	negative	-3.044	-0.258	0.0482
Cluster 2	ATP6V0D1	negative	-3.629	-0.253	0.0486
Cluster 2	RHOT1	positive	4.120	0.274	0.0435
Cluster 2	SCARB2	positive	3.655	0.288	0.0371
Cluster 2	**DNAJC13**	positive	3.040	0.310	0.0333
Cluster 2	DNAJC6	positive	3.600	0.320	0.0333
Cluster 2	PSMA1	positive	3.526	0.397	0.0105

Spearman spatial correlations between regional expression of PD-classified genes and case-control MIND strength t-maps. Genes were selected from the Allen Human Brain Atlas (AHBA) gene classification database (107 PD-specific genes); only genes showing significant spatial correlation with the t-map after Benjamini-Hochberg FDR correction are displayed (all p_FDR < 0.05). The x-axis represents normalized regional gene expression (z-scored across regions); the y-axis represents the case-control MIND strength t-value for each region. In Cluster 1 vs HC (t-values > 0, MIND increased), 5 genes survived FDR correction: 4 PLS1-negative genes (VPS26A, PSMA4, GBA2, CYCS; negative spatial correlations, indicating lower expression in regions of greater MIND increase) and 1 PLS1-positive gene (DNAJC13; positive spatial correlation).

In Cluster 2 vs HC (t-values < 0, MIND reduced), 10 genes survived FDR correction: 5 PLS1-negative genes (PSMA7, GBA2, STX3, HTRA2, ATP6V0D1; negative spatial correlations, indicating higher expression in regions of greater MIND reduction) and 5 PLS1-positive genes (RHOT1, SCARB2, DNAJC13, DNAJC6, PSMA1; positive spatial correlations). GBA2 and DNAJC13 were identified in both clusters.

These findings indicate that PD-relevant gene expression preferentially maps to regions of MIND reduction in Cluster 2, whereas in Cluster 1 the same genes map to regions of MIND increase, suggesting distinct patterns of transcriptomic vulnerability and compensatory reorganization across subtypes.

AHBA, Allen Human Brain Atlas; MIND, morphometric inverse divergence; FDR, false discovery rate; HC, healthy controls.

Supplementary Table S3. Correlation between case-control MIND strength t-maps in Cluster 1 and Cluster 2 and clinical e serum biomarkers

Cluster	Variable type	Variable	ROI	ROI name	Spearman r	p-value	p_FDR	n
**Cluster 1 — Clinical correlations**								
C1	Clinical	MDS-UPDRS	ROI_14	lh middletemporal	0.358	0.0010	0.0060	81
C1	Clinical	MDS-UPDRSME	ROI_14	lh middletemporal	0.284	0.0050	0.0300	96
**Cluster 1 — Serum biomarkers**								
No FDR-significant correlations								
**Cluster 2 — Clinical correlations**								
C2	Clinical	Onset	ROI_59	rh rostralanteriorcingulate	-0.321	0.0002	0.0032	104
C2	Clinical	Onset	ROI_25	lh rostralanteriorcingulate	-0.318	0.0002	0.0032	104
C2	Clinical	Onset	ROI_47	rh medialorbitofrontal	-0.314	0.0009	0.0096	104
C2	Clinical	Onset	ROI_11	lh lateralorbitofrontal	-0.267	0.0016	0.0128	104
**Cluster 2 — Serum biomarkers**								
C2	Serum	NfL	ROI_36	rh caudalanteriorcingulate	-0.496	0.0010	0.0160	41
C2	Serum	NfL	ROI_37	rh caudalmiddlefrontal	-0.497	0.0009	0.0160	41
C2	Serum	GFAP	ROI_25	lh rostralanteriorcingulate	-0.496	0.0010	0.0200	41
C2	Serum	GFAP	ROI_36	rh caudalanteriorcingulate	-0.468	0.0021	0.0200	41
C2	Serum	GFAP	ROI_37	rh caudalmiddlefrontal	-0.483	0.0014	0.0200	41
C2	Serum	GFAP	ROI_19	lh parstriangularis	-0.459	0.0025	0.0200	41
C2	Serum	NfL	ROI_7	lh inferiorparietal	-0.452	0.0030	0.0320	41
C2	Serum	NfL	ROI_18	lh parsorbitalis	-0.440	0.0040	0.0320	41
C2	Serum	NfL	ROI_25	lh rostralanteriorcingulate	-0.430	0.0050	0.0320	41
C2	Serum	NfL	ROI_47	rh medialorbitofrontal	-0.415	0.0069	0.0368	41

FDR-corrected Spearman correlations between regional MIND strength and clinical scores and serum biomarkers were computed, restricted to ROI showing significant case–control differences within each cluster (FWER-corrected).

In Cluster 1, two FDR-significant correlations were identified between regional MIND strength and motor severity scores. MIND strength in the left middle temporal cortex (ROI_14) correlated positively with both MDS-UPDRS total score ($r = 0.358$, $p\_FDR = 0.006$, $n = 81$) and MDS-UPDRS motor examination ($r = 0.284$, $p\_FDR = 0.030$, $n = 96$), indicating that greater structural hyperconnectivity in this region scales with increasing motor impairment. No FDR-significant correlations were observed between Cluster 1 MIND strength and serum biomarkers.

In Cluster 2, four FDR-significant negative correlations were identified between MIND strength and disease onset age in fronto-cingulate regions — specifically the bilateral rostral anterior cingulate cortex and orbitofrontal cortex (r range: −0.267 to −0.321, all $p\_FDR < 0.05$, $n = 104$). To account for potential confounding effects of disease duration, partial Spearman correlations were computed controlling for disease duration. The association remained significant in the same regions, with slightly stronger effect sizes (r_partial range: −0.306 to −0.364, all $p\_FDR < 0.05$), indicating that later disease onset is robustly associated with greater reductions in frontal MIND network organization independently of disease duration.

Additionally, 10 FDR-significant negative correlations were identified between Cluster 2 MIND strength and serum biomarkers ($n = 41$): NfL correlated with 6 ROI, and GFAP with 4 ROI, primarily involving fronto-cingulate and orbitofrontal regions, indicating that reduced structural network organization co-occurs with increased markers of axonal damage and astrocytic reactivity
